# Innovating for One Health: federal perspectives and key lessons on building integrative programs for wildlife in Canada

**DOI:** 10.1186/s42522-026-00207-6

**Published:** 2026-04-15

**Authors:** Alana A. E. Wilcox, Craig Stephen, Jennifer F. Provencher

**Affiliations:** 1 Ecotoxicology and Wildlife Health Division, Science and Technology Branch, Environment and Climate Change, Ottawa, ON Canada; 2 McEachran Institute, Nanoose Bay, BC Canada

**Keywords:** Environmental science, Research innovation, Systemic barriers, Management and conservation

## Abstract

At a national level, Canada has committed to advancing the One Health approach, recognizing the interconnection between human, animal, and environment health. One Health, however, has often focused on zoonotic diseases and their impacts on human health, limiting the scope and integration of wildlife and environment health. There is also a growing recognition of the need to develop collaborative strategies to address gaps in research, reflecting diverse expertise and priorities. To explore how One Health is perceived and can be mobilized at the federal level, we conducted a series of focus groups with staff at Environment and Climate Change Canada, discussing themes including novel and innovative program development, metrics of success, and limitations to program development and implementation. Supported by the literature, we describe the four key lessons that emerged for strengthening One Health programming: (1) adopt a settings-based approach to foster inclusivity and optimize the health of people, animals, and the environment; (2) establish a shared goal and / or mandate for a team can create a shared purpose and enhance impact; (3) identify champions and leaders who can drive innovation; and (4) bring in key experts, but maintain flexibility to ensure skills are aligned with program needs. While examples highlight innovative program development and implementation, focus group participants emphasized barriers to collaborative and integrative research on human, animal, and environment health extending beyond federal science and that require institutions to address systemic limitations on research innovation. Nonetheless, to promote optimal health outcomes, including those for wildlife and the environment, professionals can adopt integrative and interdisciplinary approaches and drive a movement from knowledge to action.

## Introduction

Independent determinants of health, including abiotic, biotic, and social factors, contribute to the health outcomes of wildlife populations [1, 2]. While individual stressors that vary in type, duration, and intensity can undoubtedly affect overall health and wellbeing, there is consensus among experts of the interconnected and cumulative effects of multiple factors on wildlife health [2]. Environmental crises such as climate change, biodiversity loss, deforestation and water scarcity, further complexify and confound the already intricate and dynamic nature of wildlife health. As such, there are growing demands for effective integrative approaches and cumulative effects management to meet the conservation targets to measure, monitor and maintain the health of wildlife populations [3].

While pathways to optimize outcomes for health are neither simple nor uniform between species or across ecosystems, the adoption of a more comprehensive approach to health - extending beyond singular species or disciplines - has been promoted as a way to tackle the complex challenges for management imposed by interacting wildlife and environment health crises. The One Health approach strives to address complex global health challenges by recognizing the integrated nature of human, animal, and environment health [4]. The framework promotes collaboration, cooperation, communication, and capacity-building, supported by several foundational principles (e.g., equity, inclusivity, equal access, parity, socio-ecological equilibrium), that have become increasingly important as One Health practices are applied to diverse settings and scenarios [4, 5]. Through the application of a holistic lens to health, a One Health framework can recognize interspecies and intergenerational health equity [6], while balancing conservation needs with social and economic factors [7, 8], and strengthening the science to policy cycle [9].

At the national level, Canada has committed to implementing the One Health approach, fostering collaboration across sectors to protect the health of animals, ecosystems, and people and promote sustainability. While informally applying One Health concepts for decades, its formal adoption into national frameworks has accelerated since the 2009 H1N1 pandemic [10] and became more structured at the institutional level during the response to COVID-19 [11]. Wildlife surveillance efforts during the COVID-19 pandemic have generated critical epidemiological information for conservation and management action and nation-wide collaborations have since been carried forward to address other wildlife zoonoses (e.g., highly pathogenic avian influenza; [12–14]). Central to Canada’s wildlife health response is the Canadian Wildlife Health Cooperative (CWHC), which plays a key role in identifying and assessing emerging health problems [15] and emerged as a key player in the development of *A Pan-Canadian Approach to Wildlife Health* (PCAWH) to protect wildlife health and preserve their ecological and economic benefits [3, 16]. Following the approval of the PCAWH in June 2018 at the ministerial level, a national 5-year strategic and operational plan was developed to support a PCAWH program, but implementation of the plan was never realized [3, 16, 17]. Nevertheless, underscoring more recent commitments to One Health in Canada, at a federal level Environment and Climate Change Canada’s Departmental Plan recently called attention to the One Health approach and continued collaboration with other federal departments, provincial and territorial governments, and Indigenous governments and organizations for research and monitoring of wildlife health issues and cumulative effects to support decision-making [18].

Despite national commitments, a gap remains in how wildlife and, more broadly, environmental research is accounted for in One Health initiatives. One Health has historically fixated on public health and the role of zoonotic disease in causing severe human illness, death, and economic burden [19–23]. On one hand, wildlife are often portrayed as a driver of negative human health impacts [24], while simultaneously, on the other, perceived as a resource available for human use (e.g., for food, materials and by-products), recreation (e.g., hunting, fishing), economic benefit (e.g., tourism, sale, licenses), and medical research. Deviating from human-centric One Health models, a settings-based approach to wildlife conservation and health focuses on protecting wildlife by considering the broader environmental, social, and economic contexts [6]. Instead of targeting individual species in isolation, the approach emphasizes the unique scenarios in a setting and the complex interactions between organisms [6]. Expanding on the settings-based approach to conservation, Stephen et al. [6] forwarded a One Health approach for wildlife conservation, which proposes that human, animal, and environment health are combined in ways unique to a setting and giving rise to multiple ways health can be experienced and assessed in a single setting. The revised approach also highlights the importance of intersectoral collaboration with shared goals to ensure that conservation efforts benefit all species and future generations equitably​ [6]. However, despite new approaches to One Health and the growing number of expert opinions calling for ideas, information, and capacities to be linked across disciplines, there is little pragmatic advice on how to mobilize collaborations into meaningful change.

With accelerating recognition of the need for new ways of acting on major global challenges related to wildlife and the environment, and increasing national-level expectation for the application of the One Health approach [18, 25], there is a need for guidance on how to respond to dynamic wildlife and environment health crises through a One Health lens and innovate in a way that bolsters science for wildlife health management and policy. Through an examination of the literature on fostering successful interdisciplinary teams and a series of focus groups with Environment and Climate Change Canada researchers, managers, and directors, our objective was to (1) explore at a national level how federal staff perceive and mobilize science to support One Health (and, in turn, wildlife health) and (2) identify key lessons that can support management for wildlife and environment health in Canada and foster innovative One Health programming.

## Methods

A series of focus groups was designed to gather information on the perception of Environment and Climate Change Canada staff on One Health and the mobilization of science in support of programs and initiatives aligned with the approach. A prospective list of participants who conduct One Health research or those managing staff involved in One Health research was drafted using institutional knowledge and internal resources at Environment and Climate Change Canada, then individuals were asked to refer other potential participants (i.e., snowball sampling). An invitation letter describing the research and focus groups was sent to all prospective participants (*n* = 25) and confirmed participants (henceforth, focus group participants) consisted on research scientists (*n* = 8), managers (*n* = 7), and directors (*n* = 5) from the Atmospheric Science and Technology Directorate, Science Policy, Planning and Partnership Directorate, Water Science and Technology Directorate, Wildlife and Landscape Science Directorate and the Canadian Wildlife Service.

Focus groups took place virtually over Microsoft Teams for up to 90 min during work hours from November 2023 - July 2024. Two sessions were conducted for research scientists and a single session each for managers and directors to accommodate schedules and create a safe space to gain insights, perceptions, and open feedback [26]. All focus groups were led by a facilitator (CS) and a co-moderator (AAEW or JFP), who handled session coordination and note-taking. Privacy and confidentiality were discussed with focus group participants at the start of each session and, at the request of focus group participants, session conversations were not recorded or transcribed. However, all focus group participants gave verbal consent for a publication without statement attribution.

Focus group participants were provided with a primer which was reviewed at the start of the focus group by CS and then a set of standardized semi-structured open-ended questions were used across the focus groups, with flexibility to allow for different question order, explore questions more in depth, and follow-up on different but relevant topics [26]. Focus group participants were asked to center responses specifically on One Health research under their administration as researchers or managers / directors. Questions included: (1) Have you succeeded in your One Health work and why was this a success? (2) Identify novel and innovative One Health projects (i.e., defined in scope and output) and programs (i.e., broad in scope and often a group of related projects managed together), what made them successful and how do we replicate that success? (3) What can be done with current funds and capacity? (4) What advice would you give to achieve game-changing innovation and progress in One Health? Managers and Directors were also asked (5) How were game-changing One Health collaborations achieved and (6) what can be or is being done in Environment and Climate Change Canada to foster or sustain innovative One Health research?

**Fig. 1:**
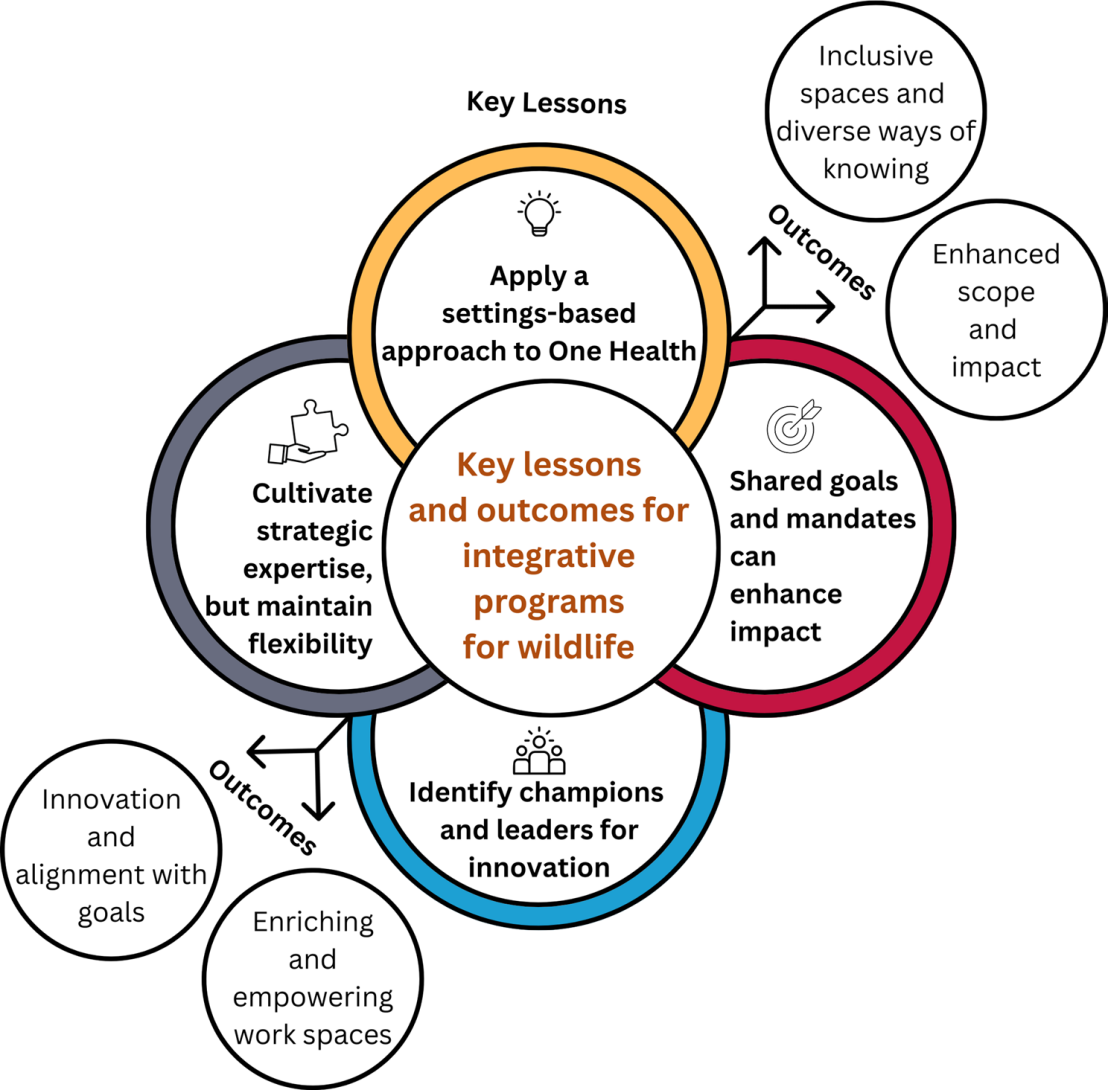
Key lessons and outcomes for the development of integrative programs for wildlife as identified in focus groups with Environment and Climate Change Canada researchers, managers and directors

After completion of the focus groups, the facilitator and co-moderators (CS, AAEW, JFP) reviewed notes and summarized the main findings, applying the principles of thematic analysis [27], and identifying themes shared by focus group participants. Four key themes (Fig. 1) were predominantly discussed among focus group participants and were then further examined alongside the literature on fostering successful interdisciplinary teams.

### Key lesson 1: A settings-based approach to One Health can foster inclusivity and support diverse ways of knowing to optimize the health of people, animals, and ecosystems

Even with animal, human, and environment health representation in the One Health framework, conventional One Health approaches often prioritize human health [28], therefore potentially overlooking complex interdependencies necessary for truly holistic health outcomes. The diminishment of animal and environment health is particularly notable when considered alongside Indigenous Knowledges, which recognize all forms of health (i.e., including, but not limited to, animal, human, and environment health) as equally significant and inherently interconnected. For example, focus group participants mentioned the Anishinaabe concept *ne’iikaanigaana* (often translated as ‘all our relations’), which highlights a worldview where human, animals, and other more-than-human beings are equal, and healthy relationships between them are critical to having good health and leading a good life [29, 30]. Even from a western-based academic approach, the focus on human health neglects how the three healths are not discrete but instead bundled together in ways that are unique to a setting [6]. In this way, management of health(s) is better aligned with the principles of ecosystem management, where management actions are focused on specific areas with clearly defined boundaries (i.e., place-based; [31]). Thus, as noted in Stephen et al. [6], a settings-based approach encourages thinking about relationships within a defined setting, targeting specific circumstances, with the ultimate aim to optimize health outcomes for all that live within the shared space. For environmental stewardship, a settings-based approach offers a more holistic framework for One Health activities that can include different ways of knowing.

Focus group participants identified the management of protected areas as an example of a settings-based approach being used to direct and structure research programs. At the federal level, Environment and Climate Change Canada is responsible for the co-management of protected areas with provincial/territorial partners and Indigenous rights-holders, including in Migratory Bird Sanctuaries and National Wildlife Areas. In Nunavut, co-management takes the form of Area Co-Management Committees, formal committees that consist of Inuit community members and Environment and Climate Change Canada staff that provide leadership in decision-making and governance on activities undertaken within the protected areas [32]. Focus group participants noted that within several of the protected areas in Nunavut, settings-based approaches have been used to explore the health of ecosystems in relation to human, animal, and environment health. In particular, work on contaminants and pathogens in harvested eider duck populations at East Bay Island in northern Hudson Bay has enhanced knowledge of shorebird ecology and capacity for Inuit self-determination [33]. The program was established to explore the population trends in eider ducks due to concerns related to overharvesting in neighboring regions, but later incorporated monitoring of pathogens and contaminants on eider and human health at the request of the community. Similarly, in the Akpait and Qaqulluit National Wildlife Areas near Qikiqtarjuaq on the east coast of Baffin Island, academic and federal collaborators have worked with the Sululiit Area Co-Management Committee since 2010 (i.e., the inception of the protected area) to track and understand multiple facets of health within the arctic ecosystem [32]. While migratory birds have been a focal point, the holistic nature of the research encompasses different ways of knowing and is directed by Area Co-Management Committees.

### Key lesson 2: Upholding a shared goal is essential for reaching meaningful One Health outcomes, but a shared mandate can enhance scope and impact

Establishing a shared goal for a team is fundamental to achieving successful outcomes in One Health research as it ensures alignment among multidisciplinary teams working toward common objectives [34, 35]. In the absence of a clearly defined goal, research initiatives risk becoming fragmented, with misaligned priorities and inefficient use of resources. In contrast, a clearly articulated shared goal serves to enhance collaboration, streamline activities, and reinforce commitment to achieving meaningful project outcomes [34]. By fostering engagement, shared goals help unify stakeholders from diverse sectors and disciplines, by promoting transparency, shared understanding, and joint accountability [36]. Shared goals also play a pivotal role in enhancing motivation and commitment, particularly when researchers see tangible opportunities for their work to be translated into practice [37]. To be effective, the goal must be clearly articulated and embedded throughout both the design and implementation phases of a project [35]. However, despite an aim of the One Health approach to create efficiency [37], effectiveness is not regularly monitored in One Health research [38]. Nonetheless, shared goals can support a robust and integrated One Health framework by guiding the generation and application of comprehensive evidence in policy and practice [38], ultimately increasing the likelihood of achieving meaningful and sustained One Health outcomes.

Within Canada, responsibilities for the pillars of One Health (i.e. agriculture, human health, environment, and wildlife) are shared between federal, provincial, territorial, and Indigenous (FPTI) partners. Thus, when working within a One Health approach, the groups that will be involved will be dictated by the topic of discussion. For example, One Health projects on avian influenza in Canada have included both federal and provincial wildlife departments, as both have mandates relating to wild birds, as well as federal and provincial agricultural experts (to include both virus regulatory authorities and farm premise regulators), and federal and provincial public health specialists. Indigenous governments and organizations across all three sectors are also involved. The diverse nature of experts engaged in One Health approaches within Canada’s response to avian influenza is demonstrated on Environment and Climate Change Canada’s approach to prioritize research and monitoring actions under an emergent highly pathogenic AIV outbreak [39]. It should be noted that while Canada is involved in many One Health initiatives, One Health is not specifically funded the FPTI level by a central office or department but rather is driven by individual groups mobilizing funding to tackle mutually beneficial projects.

The COVID-19 pandemic is recognized as an event that reinforced the One Health approach and facilitated application and implementation in research and programs [40]. The upswelling of support reflected a growing recognition of the interconnectedness between human, animal, and environment health, and the need to apply the core One Health principles (i.e., “the 4 Cs”; [4]) to improve cross-jurisdictional and multi-sectoral approaches to addressing zoonotic diseases within Canada broadly, but specifically, transmission of the SARS-CoV-2 and rapid evolution of the novel coronavirus [11]. Focus group participants within this study noted that in Canada, the pandemic accelerated the need for collaboration, coordination, and cooperation amongst FPTI departments and agencies, academic institutions, and other partners, leading to the establishment of a variety of committees and working groups, including those dedicated to surveillance and identification of potential transmission pathways within and between species [11, 41]. One Health working groups also included several sub-groups that dealt with specific issues as they emerged (e.g. Mink Sub-Working Group and the COVID-19 and Country Foods Working Group), illustrating the adaptive nature of working groups when there are shared mandates across sectors with varying engagement needed on different sub-topics.

While at the peak of the COVID-19 pandemic, partners shared a goal and mandate for national surveillance and monitoring for SARS-CoV-2 in wildlife populations, the post-pandemic period saw, at best, transformation of groups and priorities and, at worst, eschewing former One Health activities largely due to termination of COVID-specific programs, uncertain funding and financing for collaborative One Health initiatives, and a lack of an explicit cross-sectoral and cross-jurisdictional mandate. In the case of the COVID and Country Foods Working Group, the group elected to maintain its communication structure, but transition under the topic of One Health and Country Foods as it was deemed that maintaining the discussion space was beneficial to the participants [42]. For other committees, subsequent years have seen a revival of COVID-era One Health partnerships, value manifested from lessons learned, and wicked challenges being addressed through innovative multi-pronged programs, most notably to address the unprecedented international spread of highly pathogenic avian influenza [12–14]. The focus group participants highlighted a shift beginning with Environment and Climate Change Canada’s 2023-24 Departmental Plan, which under subsequent governments continues to emphasize environment, animal, and human health-related issues, requiring cross-departmental action rather than narrowly focusing on environmental concerns (Environment and Climate Change Canada [18]). Similar Departmental Plans have also taken shape in the Public Health Agency of Canada [43] and Health Canada [44]. With each department setting the scope of their One Health mandate, however, absent is the long-term - rather than program-specific - shared goal(s) for collaboration and the structures required to implement those mandates within and across departments to support One Health initiatives [45]. Therefore, the Canadian Federal Government Departmental Plans represent initial steps in the pursuit of national One Health collaborations, but careful and collaborative mandate setting that benefits multiple groups can further enhance the scope and impact of programs and policies.

### Key lesson 3: Identify champions and leaders in innovation who can create an enriching and capacity-building work environment

A dedicated champion for innovation plays a pivotal role in facilitating the acceptance and advancement of novel ideas within and between organizations. Champions may include, though is not limited to, executive level staff, trailblazers in community organizations, and leaders in Indigenous communities. By leveraging their experience and networks, such individuals can navigate uncertainty associated with transformative ideas, thereby promoting innovation and fostering institutional support. While tools should be provided to champions to support change and address systemic barriers, these individuals can also push for the creation of enriching and capacity-building work environments, advocating for resources and skill development that empowers teams to experiment and sustain innovative efforts over time. To achieve meaningful outputs from environmental research, Cooke et al. [46] highlights the importance of early engagement with teams and partners, skillfully navigating power dynamics, and frequent two-way communication with partners and stakeholders guided by high-quality, relevant plans for sharing progress. While there are numerous challenges in communicating uncertainty and research limitations [47, 48], a key factor in obtaining successful outcomes is maintaining transparency with partners and stakeholders about the strengths, risks, and boundaries of the project [46]. In this way, champions can help mitigate risk and risk aversion associated with novel and innovative ideas, thus creating a buffer for experimentation. Transparent communication that considers multiple perspectives is also a key element in maintaining strong long-term and trusting partnerships and, ultimately, translating results effectively into practice [37, 49, 50]. While champions advocate and help to secure partnerships and expand networks, leaders work directly with those frontline positions, guiding and motivating workers towards a shared goal. By operating in tandem with champions, leaders can promote on-the-ground successes and help navigate potential barriers. Therefore, the role of champions and leaders is essential for the successful integration and implementation of transformative ideas, thus opening organizations for broader change.

### Key lesson 4: Bring in key experts, but maintain flexibility to ensure skills and expertise is aligned with the program goals and organizational mandate

The successful introduction and implementation of interdisciplinary approaches in environmental sciences builds on a legacy of capacity through hiring expertise, training, and maintaining institutional memory [46], while innovative approaches further require organizational flexibility to recruit talent from diverse fields and integrate new ideas. Novel and transformative ideas require specialized knowledge and interpersonal qualities, such as open-mindedness, receptiveness to new ideas, communication skills, and the ability to adapt in team contexts [37, 51]. As such, bringing together personnel who not only possess specialized disciplinary expertise, but also demonstrate skills, like imaginative thinking, is vital to fostering effective collaboration across complex interdisciplinary settings [34]. Social identity can also have an impact on an individual’s ability to engage with novel or cross-disciplinary ideas such as perceptions of inclusion and epistemic alignment, for instance, can affect openness to new ideas and involvement and incorporation of diverse viewpoints [52]. Focal group participants noted that support for these new perspectives and ideas further underscores the importance of having a champion and leader for innovation (key lesson 3) who can promote integrative and diverse roles and help overcome organizational barriers [51]. Thus, alongside a flexible, inclusive and supportive environment, cultivating strategic expertise can support innovation.

The Northern Contaminants Program brings together groups within Environment and Climate Change Canada and other government departments, along with provincial/territorial governments, Indigenous and academic partners for both long-term monitoring and innovative research on contaminants in the environment, animals and humans in northern Canada [53]. Focus group participants explained that, taking a holistic approach, the program has a mandate to address research and monitoring of contaminants that have negative impacts on human and environment health in northern regions of Canada [54]. While core projects focus on long-term assessment of contaminant levels, shorter-term research explores the impacts on human health, animal, and environment health, as well as communication and policy outcomes. Exceptional to the Northern Contaminants Program, focus group participants noted that the network of experts with skills in natural and social science, public and environment health was established intentionally and early into the program, then maintained to support and develop cross-linkages between projects. As such, weaving together data and concepts across the public, animal and environmental fields is encouraged and promoted within the program.

### Recommendations and next steps

Moving towards a more systematic approach to One Health for wildlife and environment-related professions without massive restructuring will require comprehensive long-term programs with strong policy connections and efficient knowledge brokering to swiftly connect people and ideas. Fortunately, as seen in some of the examples described above, holistic One Health synthesis can occur when the platforms and champions are in place. As such, a structured approach that prioritizes continuous knowledge integration, assessment, and policy translation is needed to ensure coordinated action can be taken for optimal health outcomes.

Aligned with this idea, Stephen and Walzer [55] proposed a continuum of care model where interdisciplinary knowledge from different sectors is combined to guide programming and interventions, and address gaps in services and supports across the health continuum. To mitigate impacts to wildlife and environment health and promote recovery from an array of threats, a cooperative informed by continuum of care and One Health models could transform conservation and health policy by using existing data streams to anticipate emerging threats, build resilience, and work towards improving the knowledge-to-policy cycle. Further, a cooperative could link knowledge producers and decision-makers, enhance collaborations, communicate with funding bodies, and better connect wildlife, environment, and human health across disciplines and sectors, accelerating and advocating for turning innovative and evidence-based ideas and results into policy.

### Limitations

Our research has several drawbacks that should be noted when interpreting the key lessons. First, we acknowledge limitations in presenting results without transcription of focus group discussions. Despite efforts to meticulously notate conversations, the entirety of focus group participant responses and specific nuances may not have been completely captured. Second, focus groups were conducted among staff from a department within the Government of Canada and, while similar results have been reported elsewhere [3, 6, 17, 46, 56–60], the foremost lessons identified by focus group participants may differ for other federal departments and agencies or other non-governmental organizations. Findings must also be contextualized within the Canadian government structure, as well as historical and current-day relationships, networks, and transactions with multi-level governments, academics, private sector actors, and Indigenous governments and organizations. Lastly, the number of focus groups participants was relatively small and, therefore, perspectives and opinions underscoring the key lessons may not fully represent the diversity of viewpoints within Environment and Climate Change Canada or within the broader One Health community.

## Conclusion

The examples brought forward by focus group participants highlighted where Environment and Climate Change Canada wildlife and environment research and expertise contributed to innovative program development and implementation, but also articulated appreciable issues and barriers to collaborative and integrative research on human, animal, and environment health that could extend beyond the federal context. In light of evidence on declining innovation in scientific research [61], the repeated expression of systemic barriers and calls to reenvision the systems and structures currently limiting innovative wildlife and environmental research suggests that more needs to be done both within and outside these fields to address widespread and ingrained issues. Integrative and interdisciplinary approaches, such as One Health and the continuum of care model, can drive the initial steps to connect sectors and ways of knowing and, ultimately, support turning innovative, evidence-based ideas into results and policy.

## Data Availability

No datasets were generated or analysed during the current study.
